# Prevalence of helicobacter pylori among Nigerian patients with dyspepsia in Ibadan

**Published:** 2010-09-19

**Authors:** Jemilohun Abiodun Christopher, Otegbayo Jesse Abiodun, Ola Samuel Olawale, Oluwasola Olayiwola Abideen, Akere Adegboyega

**Affiliations:** 1 Department of Medicine, Ladoke Akintola University of Technology Teaching Hospital, Oshogbo, Nigeria,; 2 Department of Medicine, College of Medicine, University of Ibadan and University College Hospital, Ibadan, Nigeria,; 3 Department of Morbid Anatomy and Histopathology, College of Medicine, University of Ibadan and University College Hospital, Ibadan, Nigeria

**Keywords:** Helicobacter pylori, Histology, Rapid urease test, Nigeria

## Abstract

**Introduction:**

Determination of the true prevalence of Helicobacter *pylori ( *H. pylori*)* is difficult in a hyper-endemic area like Nigeria with use of serological tests because of their low discriminatory power between previous and current infections. The use of biopsy based methods will go a long way to mitigate this problem. We investigated the prevalence of *H. pylori* in dyspeptic patients and its relationship with gastroduodenal pathologies using gastric biopsy histology and rapid urease test.

**Methods:**

Eighty-six consecutive adult patients with dyspepsia underwent upper gastrointestinal endoscopy using forward-viewing endoscopes. Antral biopsy specimens were collected for histology and rapid urease test. Diagnosis of *H. pylori* infection was made if both or either of the tests was positive.

**Results:**

Of the 86 subjects, there were 39 (45.3%) males and 47(54.7%) females. The age range was 23 to 85 years with a mean of 49.19±13.75 years. Diagnosis of *H. pylori* was made in 55(64%) patients. Gastritis was the commonest endoscopic finding (60.5%), serious gastroduodenal pathology (gastric ulcer, duodenal ulcer and gastric cancer) were documented in only 12 (14%) patients. Thirty three (63.5%) of the 55 patients with gastritis had *H. pylori* infection while 7(58.3%) of the 12 patients with serious gastroduodenal lesions had the infection. Thirteen (72.2%) of the 18 patients that had normal endoscopic findings were *H. pylori* positive.

**Conclusion:**

The prevalence of *H. pylori* among dyspeptics using biopsy based methods is high in the South-Western part of Nigeria. It is therefore important to test and treat *H. pylori* among Nigerians with dyspepsia.

## Introduction

The discovery of Helicobacter pylori (*H. pylori*) by Warren and Marshall, in 1983 was a major breakthrough in the management of dyspepsia [1]. *H. pylori* is a gram negative, spiral, flagellated bacterium with a capability for abundant urease production which has been implicated in several upper gastrointestinal diseases that present as dyspepsia [2,3]. The organism is usually found under the mucus layer in the gastric pits in close apposition to gastric epithelial cells where it causes damage to the cells [4]. It is a major aetiological factor in chronic gastritis, peptic ulcer disease, gastric carcinoma, and gastric mucosal associated lymphoid tissue (MALT) lymphoma [2,3]. Peptic ulcer disease is now viewed as an infectious disease since eradication of *H. pylori* leads to its cure [4].

Various diagnostic tests for *H. pylori* have been developed and they can be broadly classified into invasive and non-invasive tests [4]. Invasive tests utilize endoscopic biopsy samples for histology, culture, rapid urease test (RUT) and polymerase chain reaction. All these tests have been found to have sensitivity and specificity that are well above 90% [5].

The non-invasive tests do not require endoscopy. These include urea breath test (UBT), immunoglobulin G and M serology, stool antigen test, saliva antibody test and urinary antibody test [4]. In Nigeria, the non-invasive tests are not generally available except Immunoglobulin G (IgG) serology. The value of serological tests in a hyper-endemic area like Nigeria is limited, because of their low discriminatory power between previous and current infection.

The aim of this study was to determine the prevalence of *H. pylori* among dyspeptic patients seen at the University College Hospital, Ibadan and its association with gastroduodenal pathologies using gastric biopsy histology and rapid urease test. The hospital serves as a referral centre for a substantial part of the South-Western region of Nigeria.

## Methods

The study was carried out at the Endoscopy sub-unit of the Gastrointestinal & Liver Unit, Department of Medicine, University College Hospital (UCH), Ibadan, Nigeria.

Ethical clearance was sought and obtained from the Joint University of Ibadan/ University College Hospital Institutional Review Committee. Eightysix consecutive adult patients with dyspeptic symptoms undergoing endoscopy from April 2008 to February 2009 were recruited after obtaining informed consent from them. Patients who were previously treated for *H. pylori* infection or who had received antibiotics, proton pump inhibitors or bismuth compounds in the preceding 4 weeks were excluded. Base line bio-data were obtained.

Oesophago-gastro-duodenoscopy (OGD) was performed on all the participants using Olympus (GFI-XQ20) or Pentax (FG29W) forward-viewing Oesophago-gastro-duodenoscope. Endoscopic features of each patient were recorded. Endoscopic appearance was considered normal if the mucosal was pink in colour, smooth and lustrous. Two gastric antral mucosal biopsies were taken for each of RUT and histology. A diagnosis of *H. pylori* infection was made when both or one of these two tests was positive.

**Rapid urease test (RUT)**

Two of the four antral biopsies taken from each patient were used immediately for RUT using GUT-plus by Albyn medical, Smart Medical Group,UK. GUT plus is a new generation RUT kit that consists of two dry filter paper containing urea and phenol red (a pH indicator) in a sealed plastic slide. If the urease enzyme of *H. pylori* is present in an inserted tissue sample, the resulting decomposition of urea to CO2 and NH3 causes the pH to rise and the colour of the dot turns from yellow to a bright magenta. Results were read within 3 hours after sampling according to the manufacturer’s specification. Colour change from yellow to magenta was considered a positive result while no colour change was regarded as negative.

**Histology**

The other two antral biopsies were fixed in 10% formaldehyde and transferred to the histopathology laboratory for processing. Four micron thick paraffin sections were stained with routine Haematoxylin and Eosin for detection of *H. pylori* and gastritis. Giemsa stain was also used for better yield. Slides were examined microscopically for *H. pylori* by the Pathologist. Presence of Helicobacter-like organisms was regarded as positive while absence was regarded as negative.

**Data analysis**

Data was analyzed using Statistical Package for Social Sciences, version16.0 (SPSS Inc. Chicago Illinois). Results were presented as means ± standard deviation for quantitative variables and number (percentages) for qualitative variables. Categorical variables were compared with Pearson’s Chi-square. Significant P-value was taken as <0.01.

## Results

There were 39 (45.3%) males and 47(54.7%) females. The mean age was 49.19 (±13.75) years. The age ranged from 23 to 85 years. Forty-five (52.35%) and 52 (60.5 %) patients were *H. pylori* positive with histology and RUT respectively. Fifty-five (64%) patients were positive when results of both tests were combined.

The most common abnormality at endoscopy was gastritis which was seen in 52 (60.5%) patients, followed by duodenitis 16(18.6%) and doudenogastric reflux 14(16.3%). Gastric ulcer (GU) was recorded in 8(9.3%) patients, 7(8.1%) patients had oesophagitis, while 3(3.5%) and 2(2.3%) patients had gastric cancer and duodenal ulcer (DU) respectively. Serious gastroduodenal pathologies (GU, DU and gastric cancer) were documented in only 12 (14%) patients. It is noteworthy however, that there was a considerable overlap in the endoscopic findings in these subjects as many of them with other endoscopic lesions also had gastritis.

Sixty-eight (79.1%) patients had endoscopically identifiable cause for their dyspepsia while the remaining 18(20.9%) had normal endoscopic findings. Forty -two (76.4%) patients among those with abnormal endoscopic appearance had *H. pylori* while 13 (72.2%) of those with normal endoscopic findings were positive for the organism. There was no significant difference (P-value = 0.41) between the frequency of *H. pylori* among those with endoscopic abnormality and those with normal findings. Table 1 shows the spectrum of endoscopic findings, while table 2 shows the association between *H. pylori* status and the presence of endoscopic abnormality.

The association between *H. pylori* status and various endoscopic abnormalities is depicted in Table 3. 33(63.5%) out of the 52 patients with gastritis had H . pylori infection, while 8 (50%) out of 16 and 7(58.3%) out of 12 patients with duodenitis and serious gastrointestinal lesions respectively had *H. pylori* infection. These relationships however were not statistically significant.

## Discussion

In this study, *H. pylori* was diagnosed in 64% of the patients. This is consistent with results of previous studies conducted in Nigeria and other parts of West Africa which have consistently shown a high prevalence of *H. pylori* with the use of biopsy based methods [6-11]. Previous studies conducted in various parts of South-Western Nigeria (including the University College Hospital, Ibadan) in which patients were investigated for *H. pylori* with the use of either histology or campylobacter-like organism (CLO) test showed prevalence rates of 60.5% to 73% [6,7,12] . Seroprevalence studies conducted in the same region showed prevalence rates as high as 88% to 94.5% [3,13]. These are not unexpected in a hyper-endemic area like Nigeria since serological tests cannot discriminate between previous and current infections. The sero-prevalence assay’s IgG antibody lasts for up to 3 years or more in the serum even after the organism has been eradicated.

The most common identifiable lesion at endoscopy in this study was gastritis which had a frequency of 60.5%. This is comparable to a frequency of 60% obtained in a previous study conducted in the North-Eastern part of Nigeria [9]. An earlier study conducted at the University College Hospital, Ibadan showed a frequency of 13.4% for gastritis while normal mucosal was the commonest finding with a frequency of 53% [14]. This disparity may be as a result of a change in the pattern of presentation of endoscopic lesions among patients with dyspepsia in more than two decades after the conduct of the previous study.

Our study showed that despite the high prevalence of *H. pylori* infection in the South-Western part of Nigeria, the prevalence of serious gastroduodenal pathologies (GU, DU and gastric cancer) was low as these lesions were documented in only 14% of all the patients. This is consistent with findings of previous studies conducted at the same centre and in the North-Eastern part of Nigeria [9,14].

In addition, this study showed that 63.5% of patients with endoscopic gastritis had *H. pylori* infection. This is consistent with previous studies in Nigeria that showed a high prevalence of *H. pylori* among patients with gastritis [6,9]. Also, among patients with serious gastroduodenal pathologies (GU, DU and gastric cancer) 58.3% had *H. pylori* infection. This finding corroborates the results from similar studies conducted in Nigeria and other parts of Africa [6,8,9,15]. Although the association of these lesions with *H. pylori* infection was not statistically significant, a further study involving a larger number of patients is needed to establish the true association between the infection and the lesions.

It was noted that 13 (72.2%) of the 18 subjects with normal endoscopic findings were positive for *H. pylori* and no statistically significant difference existed in the frequency of *H. pylori* infection among patients with abnormal endoscopic findings and those with normal findings . This could be explained by the very high *H. pylori* prevalence among Nigerians [13,16] and may suggest that the infection was responsible for the symptoms of dyspepsia in such patients. Hence such *H. pylori* positive patients should be treated with anti- *H. pylori* therapy.

One major limitation of this study is that it was conducted in a hospital setting and may not be a true representation of the prevalence of *H. pylori* among dyspeptics in the general population of the South-Western Nigeria, more so, that the study was carried out only in one centre. A community study is therefore desirable as this is usually more representative.

## Conclusion

This study shows that the prevalence of *H. pylori* among dyspeptic patients using biopsy based methods is high in the South-Western part of Nigeria. It also suggests that gastritis is the commonest lesion seen at endoscopy among patients with dyspepsia and that despite the high prevalence of *H. pylori* infection in the South-Western part of Nigeria, the prevalence of serious gastroduodenal pathology (GU, DU and gastric cancer) is low. Of note is the high prevalence of *H. pylori* in endoscopically normal patients. It is therefore important to test and treat *H. pylori* among Nigerians with dyspepsia.

## Competing interests

The authors declare no competing interests.

## Authors’ contributions

**ACJ**: conception and design of the study; collection, analysis and interpretation of data; and manuscript write up. **JAO**: oversight of all the stages of the research, participated in data collection and manuscript write up. **SOO**: data collection and manuscript write up. **AA**: data collection and manuscript write up OAO: histology slide report and manuscript write up.

## Figures and Tables

**Tableau 1:** Upper gastrointestinal endoscopy findings in adult Nigerian patients with dyspepsia

**Variables**		**Frequency (n=86)**
	**Present**	**Absent**
**Oesophagitis**	7(8.1%)	79(91.9%)
**Gastritis**	52(60.5%)	34(39.5%)
**Gastric ulcer**	8(9.3%)	78(90.7%)
**Gastric cancer**	3(3.5%)	83(96.5%)
**Duodenal ulcer**	2(2.3%)	84(97.7%)
**Duodenitis**	16(18.6%)	70(81.4%)
**Duodenogastric reflux**	14(16.3%)	72(83.7%)
**Normal**	18(20.9%)	68(79.1%)

**Tableau 2:** Association between *H. pylori*status and presence of endoscopic abnormality in adult Nigerian patients with dyspepsia

			**Endoscopic abnormality(n=86)**	
		**Present**	**Absent**	**Total**
**H. pylori**	Positive	42 (76.4%)	13(23.6%)	55(100.0%)
	Negative	26(83.9%)	5(16.1%)	31(100.0%)
**Total**		68(79.1%)	18(20.9%)	86(100%)

Chi-square = 0.675, df =1, P-value = 0.411

**Tableau 3:** Association between *H. pylori* status and various endoscopic abnormalities in adult Nigerian patients with dyspepsia

**Variables**			**H. pylori infection(n=86)**		
		**Positive**	**Negative**	**X2**	**P-value**
**Gastritis**	Present	33(63.5%)	19(36.5%)		
	Absent	22(64.7%)	12(35.3%)	0.014	0.906
**Serious gastroduodenal**	Present	7(58.3%)	5(41.7%)		
**lesions+**	Absent	35.1%)	26(64.9%)	0.191	0.662
**Duodenitis**	Present	8(50%)	8(50.0%)		
	Absent	47(67.1%)	23(32.9%)	1.660	0.198
**Doudenogastric reflux**	Present	8(57.1%)	6(42.9%)		
	Absent	47(65.3%)	25(34.7%)	1.336	0.562
**Oesophagitis**	Present	6(85.7%)	1(14.3%)		
	Absent	49(62.0%)	30(38.0%)	1.565	0.211*

*Fisher’s exact test, +Gastric ulcer, duodenal ulcer and gastric cancer
